# A field evaluation of a matching mechanism: University applicant behavior in Australia

**DOI:** 10.1093/pnasnexus/pgac010

**Published:** 2022-03-09

**Authors:** Pablo Guillen, Onur Kesten, Alexander Kiefer, Mark Melatos

**Affiliations:** School of Economics, The University of Sydney, Sydney, New South Wales 2006, Australia; School of Economics, The University of Sydney, Sydney, New South Wales 2006, Australia; School of Economics, The University of Sydney, Sydney, New South Wales 2006, Australia; School of Economics, The University of Sydney, Sydney, New South Wales 2006, Australia

**Keywords:** college admissions, market design, field experiment, strategy-proofness

## Abstract

The majority of undergraduate university applications in the state of New South Wales—Australia's largest state—are processed by a clearinghouse, the Universities Admissions Centre (UAC). Applicants submit an ordered list of degrees to the UAC, which applies a matching algorithm to allocate university places to eligible applicants. Applicants receive advice on how to construct their degree preference list from multiple sources including individual universities. This advice is often confusing and misleading. To evaluate the performance of the current system, we run a large sample (832 observations) online experiment with experienced participants in a choice environment that mimics the UAC application process, and in which truth telling is optimal. We vary the advice received across treatments: no advice, the UAC advice only, an instance of misleading university advice only, and both the UAC and the misleading university advice together. Overall, 75.5% of participants fail to behave in their best interest. High rates of applicant manipulation persist even when applicants are provided with the UAC's accurate advice. Students who attend nonselective government high schools are more prone to use strictly dominated strategies than those who attend academically selective government high schools and private high schools.

Significance Statement Centralized university admission systems are commonly used around the world. A “clearinghouse” uses a mathematical device called a mechanism to match applicants to degrees. Such mechanisms are commonly designed to incentivize applicants to rank degrees according to their true preferences. We run a field experiment with former university applicants where truth telling is optimal. Our findings indicate that an alarming proportion of applicants fail to understand the incentives they face. Further, we find that the accuracy and clarity of advice given by market actors, including the clearinghouse, may significantly affect applicant understanding and behavior. This can potentially translate into suboptimal assignments and subsequent suboptimal labor outcomes.

## Introduction

University admission procedures are commonly based on algorithms informed by the market design literature. Starting with Chen and Sönmez (1), there has long been concern about the ability of participants to understand the incentives they face. University admissions, school choice, and other matching problems call for the use of simple mechanisms. Participants should be able to play optimally by following straightforward advice and focus on finding out their preferences rather than strategizing. That is the logic behind recommending *strategy-proof* mechanisms^1^.

However, recent studies using the the lab-in-the-field approach pioneered by Rees-Jones and Skowronek (3) find significant manipulation rates in strategy-proof mechanisms, on the order of 25% among experienced participants. To make things worse, the use of strategy-proof mechanisms is usually impractical as it requires participants to submit a ranked list of all their possible acceptable options. Instead, participants are typically asked to submit a limited list of, perhaps, only 3 or 5 items out of 10s or 100s of possible options. The question is to what extent optimal behavior can arise under these common circumstances.

We contribute to this literature by studying the university admission system in New South Wales (NSW) by inviting experienced participants—recent high school leavers—to an incentivized online task that mimics the system administered by the official clearinghouse, the University Admissions Centre (UAC). We examine the implications of the institutional features of the mechanism—namely, restricted lists, guaranteed entry schemes, and advice provision—for applicant behavior. We design a task that simulates the UAC application process for a particular choice environment in which *truth telling—*that is, reporting degrees by order of preference—is the unique optimal strategy. Using a randomized treatment, we examine the effects of advice about listing degrees given to applicants by the UAC and a university.

Our results reveal significant comprehension problems, with 70%–80% of participants behaving suboptimally depending on the kind of advice provided. Furthermore, we find that, while this behavior is significantly affected by misleading advice given by universities, it is almost completely impervious to accurate advice given by the administrators of the mechanism.^2^

Each year, over 40,000 graduating high school students in NSW apply to university.^3^ Applicants submit an ordered preference list of up to 5 degrees for which they wish to be considered.^4^ To generate offers to students, the UAC applies a matching algorithm that accounts for a student's individual assessment score and the university-determined, degree-specific, entry cut-off scores.

While the UAC admissions system appears similar to a typical college admissions problem (6), universities can influence student applications through an additional channel. To limit the uncertainty faced by applicants, universities often grant applicants “guaranteed entry” options. In essence, such schemes involve a university preannouncing an upper limit on their entry cut-off for a particular degree. This innovative feature of the UAC system can be viewed as a centralized, algorithmic embodiment of “early decision” schemes used by over 2/3 of top colleges in the United States (12) that admit stud ents through a decentralized system.^5^Under the UAC algorithm, if an applicant includes a guaranteed entry degree in her preference list, this implies that she will not be considered for any degree that she has listed lower on her list provided that she attains the preannounced entry score.^6^ In this sense, guaranteed entry options are similar to ‘district school priority’ (1, 13).^7^

While guaranteed entry options help reduce uncertainty at the time of preference submission, they can be used suboptimally by applicants. Specifically, in choice environments where truth-telling is optimal, applicants might suboptimally list guaranteed degrees above other degrees that they prefer and might be eligible for.

Additionally, prior to the offer process, applicants often seek advice on how to list their degree preferences; from the UAC, universities, or other parties such as school careers advisors or parents. The UAC provides applicants with the following standard advice:

*“List your ‘dream preference’ at number one but follow that with realistic preferences. At the bottom of the preference list you should include one or two ‘safe’ options to ensure that you get an offer”* (14).

On the other hand, universities often provide advice that conflicts with the UAC advice:

*“To be offered a place in a guaranteed entry course* ^8^ *, list the course as your first preference when you apply”* (Major University A, 2018).

“*The only way we can guarantee you a place is if you have the guaranteed entry (selection rank) and you have the degree listed as your highest eligible preference*.” (Major University B, 2018).

The General Manager of Marketing and Engagement at the UAC describes the difficulty of dealing with students who face such conflicting advice:

*“We get hundreds, if not thousands, of applicants every year who contact us because they are unsure about how to order their preferences. We tell them that they don't need to have their guaranteed entry degree as their first preference unless it really is the one they want the most, but they often say they will put it first anyway, just to be on the safe side. It's frustrating in a way, but you can also see where they're coming from. The stakes for them are high. They are far more concerned about missing out on an offer than they are about using the system to their own advantage.”* (Kim Paino, General Manager, Marketing and Engagement, UAC).

In our design, participants are able to apply for up to 5 degrees from a choice set of 6 degrees. They are informed that they have guaranteed entry to the degree with the fifth highest payoff. Since truth telling is the unique optimal strategy,^9^ this degree should be listed fifth. Participants who list this degree higher than fifth are described as exhibiting *Guaranteed Entry Bias (GEB)*. We randomly assign participants to 1 of 4 treatment groups. All groups receive a common set of instructions regarding how to submit their degree preference lists. The ‘No Advice’ group receives only these instructions. The ‘UAC Advice’ group additionally receives the accurate UAC advice quoted above while the ‘University Advice’ group receives the misleading university advice quoted above. The fourth—‘Combined Advice’—group receives *both* the UAC advice and the university advice.^10^

Despite truth-telling being the optimal strategy, a staggering 75.5% of participants across treatments manipulate in some way. We refer to participants who fail to tell the truth as exhibiting *Sub-optimal Ordering*. In particular, 55.8% of participants exhibit *GEB*. This rate is higher than that for another suboptimal strategy—*Including Degree 6—*that involves participants including the least preferred degree (Degree 6) in their list, despite it being dominated by all the other 5 degrees including the guaranteed entry degree. These results suggest a widespread misunderstanding of the UAC application process.

While rates of *Sub-optimal Ordering* and *GEB* are high across all treatment groups, they are statistically significantly higher for the University Advice and Combined Advice groups. The effect of university advice is not surprising; universities supply guaranteed entry opportunities which are highly valued by applicants. As such, applicants list the guaranteed entry degree higher than they should. However, even when the university advice is combined with the UAC advice (which performs significantly better on its own), the combined effect is almost indistinguishable from the pure University Advice effect. This suggests that accurate, albeit somewhat complicated, advice fails to mitigate the impact of misleading (but straightforward-sounding) advice.

We also examine how demographic factors interact with advice received to influence the degree preference list applicants submit. We find statistically significant differences based on high school type controlling for advice treatment and other factors. Participants who attended nonselective (i.e. comprehensive) government high schools exhibit the highest rates of *Sub-optimal Ordering*. Participants who attended selective government high schools exhibit the lowest rates. These results align with a series of studies finding higher rates of suboptimal behavior for lower ability applicants (3, 15, 16). However, there is a lower rate of *GEB* for private school students, suggesting that greater access to (accurate) advice may also play a role. These results suggest that a misunderstanding of the UAC application process may be more prevalent among some demographic groups than others.

## Institutional context

The UAC processes applications for the majority of undergraduate university degrees in NSW. Applicants submit an ordered list of up to 5 degrees that they wish to be considered for. Based on these lists, the UAC generates degree offers on behalf of universities.^11^

The UAC Mechanism uses 3 pieces of data: the degree preference lists submitted by applicants, each applicant's selection rank for a degree, and a university's Lowest Selection Rank (LSR) for a degree.

The UAC's offer algorithm is a recursive process that runs as follows for a given applicant:

*Step 1:* consider the applicant's first-listed degree. Is their selection rank above the selection rank cut-off? If yes, make an offer and stop. If no, proceed to the next step.
*Step k:* repeat Step 1 for the applicant's *k*-th listed degree.

If no offer is made after considering the last listed degree, then no offer is made.

An applicant's selection rank is the sum of her Australian Tertiary Admissions Rank (ATAR) and any ‘adjustment factors’ she is eligible for. The ATAR is a standardized performance ranking calculated on the basis of assessment results in the last year of high school.^12^ It is designed to allow universities to compare applicants on the basis of academic achievement. Applicants know their ATAR when submitting their ranked lists. Each university submits to the UAC a LSR for each degree. However, adjustment factors can increase an applicant's selection rank above their ATAR. The application and calculation of adjustment factors differs across universities and even across different degrees at the same university. This means an applicant may have a different selection rank for every degree that she applies for.^13^

Applicants are ordinarily not informed of the LSRs that universities submit to the UAC. Instead, they only know the LSRs from the *previous year* and are told that these are merely a guide (18).

Recently, some universities have started marketing ‘guaranteed entry’ to particular degrees. This is not a separate application process. Instead, some universities publish a Selection Rank which ‘guarantees’ an applicant an offer of entry to a degree provided that they: (i) include the degree in their list; (ii) achieve a Selection Rank for that degree that meets or exceeds this ‘guaranteed’ Selection Rank; and (iii) do not receive an offer to a higher listed degree.

Essentially, these universities preannounce an upper limit on the LSR for particular degrees, for *the current year*. They are still able to set the actual LSR lower than this.

Accordingly, an applicant who knows that they satisfy requirement (ii) no longer faces uncertainty about whether they are eligible to be made an offer to the guaranteed entry degree when ranking degrees. An applicant knows (ii) is satisfied if their ATAR (which they know when ranking degrees) exceeds the ‘guaranteed’ Selection Rank. Alternatively, an applicant might know that they are eligible for adjustment factors that will raise their Selection Rank above the ‘guaranteed’ Selection Rank.

Comparing this with nonguaranteed entry degrees, an applicant who knows that their Selection Rank for a degree is above the LSR from the *previous year*, still faces uncertainty about whether the LSR for *the current year* may be higher.

## Underlying theory and experimental setup

A finite set $A$ of applicants apply for admission to a degree from a finite set $C$ of degrees, where particular universities administer different degrees. Applicants have strict preferences, i.e. complete, transitive, and antisymmetric orderings, over degrees. We represent a generic ordering as $( a ) = c_{1}\;,c_{2},\;c_{3},\;a,\; \ldots $, denoting that applicant *a* prefers degree $c_{1}$ first, then degree $c_{2}$, then degree $c_{3}$, and then prefers being unmatched to being matched to any other degree besides these.

For each degree administered, universities hold strict preferences over individual applicants based on their selection ranks. Let $SR_a^c\;$denote the selection rank of candidate *a* for degree *c*. Let $P( c )$ denote the strict preference and $R( c )$ the weak preference of degree *c*. Thus, ${a_1}R( c )\;{a_2}$ if and only if $SR^{c}_{a_{1}} \ge SR^{c}_{a_{2}}$ for any pair of applicants ${a_1}$ and ${a_2}$ and any degree *c*. A typical preference ordering may be denoted as $P\;\;( c ) = {a_1}\;,{a_2},\;{a_3},\;c,\; \ldots $, meaning that the university administering degree *c* most prefers applicant ${a_1}$, then applicant ${a_2}$, then applicant ${a_3}$, and then prefers having any remaining places unfilled to being filled by other applicants. Such preferences are induced by the cut-off scores submitted to the UAC by each university for each degree.

A (university admissions) *problem* specifies the preferences of each applicant and each degree. A matching is a mapping from *A* to *C*⋃*A* such that each applicant is assigned to at most 1 degree. A *mechanism* chooses a matching for each problem. A mechanism is *strategy-proof* if truth telling is a dominant strategy for each applicant.

The UAC mechanism can be viewed as a simple version of the well-known Boston Mechanism (BM). The key difference between the UAC application process and most uses of the BM, is that universities are not bound by admissions caps nor, for all intents and purposes, do physical capacity constraints bind in practice. Therefore, in our setting, the BM is equivalent to the celebrated Deferred Acceptance (DA) mechanism (17). However, the UAC mechanism is not strategy-proof because applicants can only include a maximum of 5 degrees in their ordered lists. If an applicant wishes to apply to 5 or less degrees, then they can still report truthfully. However, if they wish to apply to more than 5 degrees, then they need to make trade-offs about which degrees to include and exclude (18).^14^

Our experiment studies a particular example for which the UAC mechanism is strategy-proof. In our example, the set of available degrees is restricted to 6 . That is $C\; = \lbrace {c_{1},\;c_{2},\;c_{3},\;c_{4},\;c_{5},\;c_{6}} \rbrace\;$. These degrees have associated monetary payoffs, with $c_{1}$ having the highest payoff, $c_{6}$ having the lowest payoff, and payoffs decreasing monotonically between $c_{1}$ and $c_{6}$. Assuming applicants prefer more money to less, this induces a common preference ordering, $P\;\;( a ) = c_{1}\;,c_{2},\;c_{3},\;c_{4},\;c_{5},\;c_{6},\;a$. While applicants know the previous year LSRs for each degree, they are told these should only be used as a guide, as they may change in the current year.

Further, applicants are told that they have guaranteed entry to degree $c_{5}$. This means that, if they list $c_{5}$ first, they know that they will be made an offer to $c_{5}$ provided that they meet or exceed its associated LSR. Similarly, if they list $c_{5}$ second, they know they will be made an offer to $c_{5}$ if they meet the LSR hurdle and are not made an offer to their first listed degree, and so forth. The unique optimal ordering in this case is to list the degrees in order of *true* preference excluding $c_{6}$, specifically $( {c}_{1},\;c_{2},\;c_{3},\;c_{4},\;c_{5} )$. This is because, under the UAC algorithm, the possibility that an applicant is made an offer to a particular degree does not depend on where it is listed, except that she will miss out on an offer to a lower listed degree if she accepts an offer to a higher listed degree. Given this particular choice problem, we now proceed to identify some dominated degree orderings.

### GEB

Conditional on 1 of $\lbrace {c_{1},\;c_{2},\;c_{3},\;c_{4}} \rbrace$ being attainable, it is a dominated strategy to list $c_{5}$ higher than in the fifth position. Where it is listed higher than fifth, the preference ordering will be said to exhibit *GEB*; this is analgous to the ‘District School Bias’ problem.^15^ Listing $c_{5}$ higher than fifth does not increase an applicant's chances of being made an offer to $c_{5}$. This is because, if $c_{5}$ is listed anywhere, an offer is generated for it unless an offer is generated for a degree listed higher by the applicant. Hence, listing $c_{5}$ higher than fifth, decreases the likelihood of being made an offer to at least 1 degree from the set {$c_{1},\;c_{2},\;c_{3},\;c_{4}\rbrace $, all of which are strictly preferred to $c_{5}$. Moreover, listing $c_{5}$ higher than fifth, and subsequently accepting an offer for $c_{5}$, ensures that the applicant will not be made an offer to any degree listed below $c_{5},$ at least 1 of which must come from the preferred set {$c_{1},\;c_{2},\;c_{3},\;c_{4}\rbrace $.

### Include Degree 6

Including the least preferred degree, $c_{6}$, is also a dominated strategy. The guaranteed entry degree, $c_{5}$, is strictly preferred to $c_{6}$. If an applicant lists $c_{6}$ above $c_{5}$, then they decrease the likelihood of being made an offer to $c_{5}$. If an applicant lists $c_{6}$ after $c_{5}$, then they must have left out at least 1 degree from the set {$c_{1},\;c_{2},\;c_{3},\;c_{4}\rbrace $, all of which are strictly preferred to $c_{6}$.

## Experimental sample

The UAC drew random samples from the population of 2018 NSW Grade 12 students, who applied for 2019 university entry through the UAC. We generated a unique survey link to *Qualtrics*—the software program used to run the survey—for each potential applicant and provided these to the UAC for email distribution.

Participants were first shown a consent form, which required them to acknowledge their understanding of the survey, including the terms of the participant information statement. The survey comprized 2 parts: a matching task, in which participants were asked to apply for hypothetical university degrees, followed by a series of questions to elicit relevant background information about the participant.

Across 7 waves, the UAC emailed 14,500 former applicants, who were randomly selected from the population of approximately 40,000 applicants. That is, approximately 36% of applicants were contacted. In total, 832 people completed the survey; a response rate of 5.7%.

In terms of gender, 62% of our participants are female vs. 55% in the population; see Table S1 (Supplementary Material) in the Online Appendix for the summary statistics related to gender. In terms of the type of high school, 34.5% attended a government nonselective high school, 22.5% a government selective high school, and 43% a private high school; see Table S2 (Supplementary Material) in the Online Appendix for the summary statistics related to school type. By contrast, in the population 43% of applicants attended a nonselective public high school, 7% a selective public high school, and 50% a private high school. Therefore, our sample is biased toward female participants and participants from selective public high schools, an issue to which we return to later.

Participants were also asked their birth order among siblings (if any); see Table S3 (Supplementary Material) in the Online Appendix for the relevant summary statistics. This question aimed to determine whether having a sibling who had previously experienced the UAC application process might influence a current applicant's approach to constructing their preferred degree list. In our sample, 85% of participants reported being either first in order of birth among siblings (54%) or second in birth order (31%); that is, most of our participants are the first in their family to experience the current UAC application process either because they are the first-born or an only child.

Finally, participants were asked about the sources of advice they accessed when applying for University entry in 2018; see Table S4 (Supplementary Material) in the Online Appendix for the summary statistics related to this so-called “prior advice”. Most participants accessed multiple sources of data. The UAC website, school teachers, and school careers advisors were most commonly used (by up to 75% of participants), followed by University websites and open-days (by approximately 40% of participants).

## Detailed experimental design

In the matching task, participants were presented with a choice environment simulating a main offer round. That is, participants could apply for up to 5 out of 6 degrees with guaranteed entry to their fifth most-preferred degree. As in other matching experiments monetary incentives were used to induce a particular preference ordering (1, 5, 8). Under the assumption that participants preferred more money to less, they should have most preferred the highest paying degree, and then preferred the other degrees in order of decreasing monetary payoffs.

This approach overcomes a key challenge discussed in the empirical matching literature, the inability to observe subjects’ preferences.^16^

Participants were informed that the task was similar to applying through the UAC. They were then given a set of common information. This included: a common ATAR, a choice set of 6 degrees, information about those degrees, information about the procedure to be applied, and that they had guaranteed entry to the degree paying the fifth highest amount.

Each participant was informed that they had been awarded an ATAR of 80.00 for the purposes of the task. This ATAR was chosen to roughly approximate that of an average applicant, and hence make the task more familiar for most applicants.

Participants were presented with the first 3 columns in Table 1, listing the 6 available degrees. Consistent with official UAC communications (14), the LSR was described as the prior year's Selection Rank cut-off for admission; merely a guide for the current year. The ‘amount paid if made an offer’ was described as the amount a participant would be paid—in addition to a $5 participation fee—if she received an offer to the associated degree.

**Table 1.** Degree information with offer eligibility probabilities.

**Degree numerical code**	**LSR**	**Amount paid if made an offer**	***Probability of obtaining an offer (not displayed to participants)***
42055	85.00	10.00	*0.10*
19959	82.00	9.00	*0.30*
56769	81.00	8.00	*0.50*
65028	80.50	7.00	*0.75*
73027 (guaranteed entry)	79.00	6.00	*1*
82747	78.00	5.00	*1*

This table reports the numerical codes of each degree presented to the subjects and the corresponding LSR, payment, and a subject's ex ante probability of getting an offer from that degree.

Participants were then informed that the offer-making procedure simulated the UAC procedure; that the further their ATAR below a degree's LSR, the lower their chance of being made an offer to that degree; and that they had guaranteed entry to the degree with the fifth highest payoff. This last was explained to mean that the university offering $c_{5}\;$had informed them that their selection rank (in this case, their ATAR plus adjustment factors) was above the LSR for $c_{5}$. Degrees were shown to participants with a numerical code, as in Table 1. Participants were then asked to number a maximum of 5 boxes, corresponding to the degrees they wished to be considered for. These boxes were presented in a random order to each participant. This ensured that participants simply ordering the first 5 degrees from 1 to 5, would be unlikely to order the degrees in the uniquely optimal way.

Keeping this information consistent across participants facilitated comparison of their ordering decisions. Participants were randomly assigned to 1 of 4 groups. The ‘No Advice’ group only received the common information. The ‘UAC Advice’ group additionally received advice about how to list preferences from a UAC publication (18). The ‘University Advice’ group additionally received the ‘Major University A’ advice quoted in the Introduction and used by a major NSW university on their website and in publications at the time when the participants were applying for university entry. The ‘Combined Advice’ group received both pieces of advice. To mitigate potential experimenter demand effects, participants were told “UAC gives the following advice…” or “The university which runs degree 73207 gives the following advice…”.

Each degree was assigned a probability (not communicated to participants) that a participant's selection rank would exceed its LSR; that is, that a participant would be eligible to be made an offer to it. These probabilities were designed to simulate the likelihood that a participant with an ATAR of 80.00 would have a selection rank above the listed LSRs from the previous year. There are 2 reasons why an applicant with an ATAR below the published LSR, may in fact exceed the cut-off for the current year. First, she may be eligible for adjustment factors. Second, the LSR may be lower than in the previous year. Consistent with common practice we did not inform participants of their adjustment factor eligibility.

We calculated payments by applying the UAC algorithm to degree lists in conjunction with the eligibility probabilities. We coded this procedure into *Qualtrics* generating a random number between 1 and 1,000 for each participant (but unknown to them). We then displayed a message to the participant with their calculated payment amount based on how they had ordered their degrees and if the random number was within certain bounds.

After the matching task, participants were asked a series of demographic and background questions relating to prior advice received (see the Appendix). Finally, participants were informed how much they were eligible to be paid and were asked to provide their mobile phone number and register for online payment.

## Results

Table 2 provides a summary of observed manipulation by advice treatment group. Each column provides the proportions of participants who were part of the listed treatment group, whose submitted degree preference list exhibits either *GEB*, or *Include Degree 6*, or any type (or types) of *Sub-optimal Ordering*.^17^ The observed rate of suboptimal ordering across treatment groups ranged from 70.5% for the UAC Advice group to 80.5% for the University Advice group. The rate of suboptimal ordering among participants who received no advice was 71.6%. That is, among participants who were reliant on what they remembered about the UAC application process, 71.6% did not tell the truth despite it being optimal to do so.

**Table 2.** Suboptimal ordering outcomes by advice treatment group.

	**No Advice (%)**	**UAC Advice (%)**	**University Advice (%)**	**Combined Advice (%)**
***Any Sub-optimal Ordering***	71.6	70.5	80.5	79.0
***GEB***	50.5	47.0	63.8	61.4
***IncludeDegree 6***	42.8	44.5	47.1	50.5
Number of observations in the treatment group	208	200	210	210

This table reports the proportion of participants in each treatment group (including the No Advice baseline group) who submited preferred degree lists exhibiting *GEB*, *Include Degree 6* or *Any Sub-Optimal Ordering*. Note that an applicant's preferred degree list may exhibit more than 1 of these types of suboptimal ordering.

Table 2 also shows that *GEB* and *Include Degree 6* are commonly observed across all treatment groups. The rate at which *GEB* is observed ranges from 47% of submitted preference lists in the UAC Advice group up to 63.8% in the University Advice group. The rate at which *Include Degree 6* is observed ranges from 42.8% of submitted preference lists in the No Advice group up to 50.5% in the Combined Advice group.

In order to formally estimate the impact that advice provided to applicants has on the degree orderings they submit to the UAC, we separately regress each of the dominated degree orderings identified earlier—namely, *GEB* and Including $c_{6}$—as well as an indicator of any suboptimal ordering, on indicators of the UAC, University, and Combined Advice treatment groups. Demographic and prior advice variables are included as covariates. As such, our results are expressed relative to the excluded ‘No Advice’ base case in which an applicant does not receive any advice on how to list their degree preferences either from the UAC, or a University (or both).

The OLS regressions estimated take the following form:

(1) \begin{eqnarray*} GE{B_i} &=& \beta _0^{GEB} + \beta _1^{GEB}UAc_{i} + \beta _2^{GEB}UN{I_i} + \beta _3^{GEB}COMBINE{D_i} \\ &&+\, {\alpha ^{GEB}}X_i^{\prime} + {\varepsilon _i}, \end{eqnarray*}

(2) \begin{eqnarray*} Include\;Degree\;{6_i} &=& \beta _0^{ID6} + \beta _1^{ID6}UAc_{i} + \beta _2^{ID6}UN{I_i} + \beta _3^{ID6}COMBINE{D_i} \\ &&+\, {\alpha ^{ID6}}X_i^{\prime} + {\nu _i}, \end{eqnarray*}

(3) \begin{eqnarray*} Any\;\;Sub - optimal\;Orderin{g_i} &=& \beta _0^{SoO} + \beta _1^{SoO}UAc_{i} + \beta _2^{SoO}UN{I_i} \\ &&+\, \beta _3^{SoO}COMBINE{D_i} + {\alpha ^{SoO}}X_i^{\prime} + {\eta _i},\\ \end{eqnarray*}

where $GE{B_i} = \;1$, if applicant *i*’s submitted degree preference list exhibits *GEB* and 0 otherwise; $Include\;Degree\;{6_i} = \;1$, if applicant *i*’s submitted degree preference list includes Degrees 6 and 0 otherwise; $Any\;Sub - optimal\;Orderin{g_i} = \;1$, if applicant *i*’s submitted degree preference list exhibits any form (or multiple forms) of manipulation and 0 otherwise; $UA\;\;c_{i} = \;1$, if applicant *i* is part of the UAC Advice group, $\;0$ otherwise; $UN\;\;{I_i} = \;1$, if *i* is part of the University Advice group, $\;0$ otherwise; $COMBINE{D_i} = \;1$, if *i* is part of the Combined Advice group, $\;0$ otherwise; $X_i^{\prime}$ is a vector of demographic and prior advice variables; and ${\varepsilon _i}$, ${\nu _i}$, and ${\eta _i}$ are random error terms.

Table 3 reports the coefficient estimates associated with each treatment group for the OLS regressions specified in Equations (1)–(3) above.

**Table 3.** Regression coefficients for suboptimal degree preference listing by advice treatment.

	***GEB***	**Include Degree 6**	**Any Sub-Optimal Ordering**
**UAC Advice**	−0.031 (0.05)	0.023 (0.05)	−0.008 (0.046)
**University Advice**	0.131*** (0.049)	0.034 (0.049)	0.083** (0.041)
**Combined Advice**	0.113** (0.05)	0.088* (0.049)	0.081* (0.042)

Notes: SE in parentheses.

Asterisks indicate significance levels: * = *P*-value < 0.1; ** = *P*-value < 0.05; and *** = *P*-value < 0.01.

No Advice is the baseline treatment in each case.

Table 3 shows that, on average, and relative to the No Advice baseline, degree preference lists submitted by applicants who receive only the misleading University Advice are 13% more likely to display *GEB* (statistically significant at the 1% level). Similarly, applicants who receive both the misleading University Advice and the accurate UAC advice are 11% more likely to submit degree preference lists that display *GEB* than if they had received no advice at all (statistically significant at the 10% level). When applicants only receive the accurate UAC advice, the degree preference lists they submit are 3% *less* likely to display *GEB* compared to the No Advice baseline, but this estimate is not statistically significant.

Table 3 also shows that both the accurate UAC and the misleading University advice *on their own* have a positive, but statistically insignificant, impact on applicants including Degree 6 when constructing their degree preference lists (relative to the No Advice baseline). On the other hand, when the UAC and University advice are combined, applicants are 8.8% more likely (significant at the 10% level) to sub-optimally include Degree 6.

Table 3 reveals that advice can significantly impact the propensity of applicants to submit degree preference lists that display any (including multiple types of) suboptimal ordering. Applicants receiving only the misleading University advice are 8.3% *more* likely to submit a suboptimal ordering (significant at the 5% level) than if they had received no advice at all. Applicants receiving both the University and UAC advice together, are 8.1% *more* likely to submit a suboptimal ordering (significant at the 10% level) relative to the No Advice baseline. On the other hand, applicants who received only the UAC advice were 0.8% *less* likely to submit a suboptimal ordering, albeit this was statistically insignificant.

The results reported in this section suggest up to 3 times more manipulation than observed in previous studies. This is in spite of the fact that a high proportion of the participants in our study attended (academically) selective government schools; our sample is biased toward higher ability students when compared to the population at large.

Our results indicate a poor general understanding of how the UAC offer-making process works and that university applicants may be susceptible to *GEB*. Applicants may believe that they risk missing out on their Guaranteed Entry offer unless they place it high on their degree preference list. This may also reflect a fear that if they miss out on the Guaranteed Entry degree, they may not receive any offer. Misleading university advice, implying that eligibility for the Guaranteed Entry degree requires listing it first , may be exacerbating applicant suboptimal behavior.

**Fig. 1.**
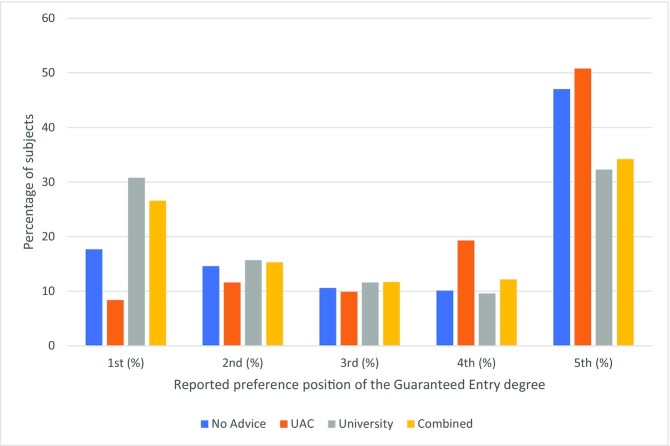
Ordering of the guaranteed entry degree by treatment group.

The UAC Advice group appeared least susceptible to *GEB*; that is, listing the guaranteed entry degree higher than in the fifth position. Figure 1 shows where participants listed their guaranteed option. While the fifth position is modal across all treatments, it only surpasses 50% under the UAC advice. This emphasizes just how compelling the University advice is: the proportion of participants ranking their guaranteed option first increases from 8% in the UAC Advice treatment to above 30% in the University Advice treatment. Providing both pieces of advice only marginally offsets the deleterious impact of the University Advice.

The vertical axis shows the *percentage* of subjets reporting their Guaranteed entry degree in positions 1st–5th.

We also examine the impact of demographic factors and prior advice, controlling for treatment group. We report these estimates from regressions (1)–(3) in Table S5 (Supplementary Material) in the Online Appendix.

All else equal, we estimate that participants from nonselective public high schools were 6.9% and 8.2% more likely to exhibit *GEB* than participants from private and selective high schools, respectively. These estimates are significant at the 10% level. We also estimate that participants from nonselective government schools were 4.8% and 17.3% more likely to exhibit *Sub-optimal Ordering* than students from private and selective government schools, respectively. The estimated difference between nonselective and selective government school applicants is significant at the 1% level. We observe no significant variation in *Include Degree 6* across school type.

These results suggest that students from nonselective government schools understand the UAC offer making process the least. This may be due to private and government selective schools investing additional resources in advice provision, such as careers advisors. Or, as earlier studies suggest, low ability students may be most affected by mechanism misunderstanding (19). Regardless, these findings suggest that students from relatively disadvantaged schools may be more exposed to the consequences of sub-optimal ordering.

## Discussion

We have examined the university admissions system in NSW, Australia, which is based on a central clearinghouse with innovative features including guaranteed entry schemes that resemble Early Admission programs in the decentralized college admission system in the United States. However, we caution that conflicting advice can significantly undermine the intended benefits of such schemes.

We construct a choice environment in which truth telling is the unique optimal strategy. Our results indicate widespread applicant misunderstanding of the UAC matching mechanism: the majority of applicants list the degree for which they have a guaranteed offer higher than is optimal.

The proportion of participants behaving suboptimally in our experiment (above 70% across treatments) is extremely high particularly since they are already familiar with the system. University admissions systems carry high stakes. For example, the salary expectations for a *first job* in dentistry and pharmacy in Australia are $80k and $39k, respectively (20). An applicant with a guaranteed offer in pharmacy, who prefers (and also qualifies) to study dentistry, could easily miss out on studying the latter implying a potentially significant reduction in lifetime income and employment satisfaction.

Our study also assesses the effects of advice given by a market operator and market actor, in a field setting. We find that misleading advice given by a market actor significantly increases manipulation and that accurate, yet complicated, advice provided by the market operator does not significantly reduce, much less neutralize, this impact. This suggests that market operators need to pay close attention to the advice being given by other market actors. More particularly, understanding the implications of Guaranteed Entry in a system like that managed by the UAC is important. Calsamiglia et al. (13) find that priority entry biases are more prominent in constrained choice environments; in the presence of a constraint, applicants are required to make trade-offs between degrees that they want to apply for.

We also find evidence of rates of suboptimal ordering and *GEB* being associated with school types. This suggests that mechanism misunderstanding may be correlated with demographic factors disadvantaging particular groups. These findings may provide a basis to make changes to the current application process and the advice given to applicants.

## Materials and methods

The project was approved by the University of Sydney Human Ethics Committee as protocol 2019/544. The experimental method is described in the Experimental Sample and Experimental Design sections. Further materials can be found in the Appendix, namely, omitted summary statistics and regressions; the email sent to prospective participants; survey questions; and the participant information statement.

## Supplementary Material

pgac010_Supplemental_File

## Data Availability

All the data collected in this research is publicly available at https://www.dropbox.com/s/2hce9bjapj4b7dj/Article%20Data.csv?dl=0.

## Footnotes

1 A mechanism is *strategy-proof* if it is in the best interests of a participant to truthfully report her preferences; i.e. in the terminology of game theory, truthfulness is a (weakly) dominant strategy. When referring to strategy-proofness in this paper, we are implicitly assuming that participants’ preferences are determined only by their final assignment to a university degree. However, some recent research argues that participants in matching markets are more rational than they appear to be. Reporting a ‘suboptimal’ preference ranking might then be explained by loss aversion; see, for example, (2).

2 Guillen and Hing (5) use stylized advice in a laboratory experiment. Ding and Schotter (6) and Ding and Schotter (7) use participants’ cheap talk and intergenerational advice in laboratory settings. See also (8, 9) for other examples of laboratory experiments that aim to embed realistic features of actual implementation.

3 See Chen et al. (10) for a summary of the current directions in market design and Hakimov and Kübler (11) for a recent review of the school choice literature.

4 Some applicants, most notably international students, apply directly to universities bypassing the UAC. This paper relates only to the vast majority of applicants (mostly domestic school leavers) who apply via the UAC.

5 In the United States, Early Decision Programs represent a mutual commitment to enrol between a student and a college, provided the student meets specific admissions criteria. When a student includes a guaranteed entry school in her preference list, she may still be eligible for colleges she ranks higher.

6 Like the UAC, the Universities and Colleges Admissions Service (UCAS) in the United Kingdom also offers guaranteed admission. In the UCAS, students can rank up to 2 schools, but they cannot rank a second school if their first is guaranteed.

7 Note, however, that district school priority is granted by school districts, not strategically determined by individual schools.

8 In Australia, the word ‘course’ is synonymous to ‘degree’

9 This design choice makes it possible for us to study the effect of guaranteed entry bias in isolation from the effect of strategizing due to constraints on preference lengths.

10 To mitigate potential experimenter demand effects, we expressly attributed the advice to UAC or the university running the guaranteed entry course; extracted advice in quotation marks to distinguish it from the surrounding instructions; and labeled this as ‘advice’. The (experienced) participants were also told to submit preferences as if applying through UAC, and were free to further research UAC procedures.

11 In fact, offers are made across various ‘offer rounds’ ahead of which applicants can adjust their ranked lists of degrees. However, the majority of offers are made in the 2 rounds following the release of student exam results. Our study simulates what occurs in 1 of these rounds.

12 These results measure student performance in both in-school assessments and external examinations.

13 Some common reasons for the application of adjustment factors include experiencing disadvantage during the last 2 years of school or notable performance in subjects related to the degree applied for. Applicants are often unaware of their eligibility for, as well as the size of, any adjustments.

14 The exception to this is if an applicant is considering applying to more than 5 degrees but has guaranteed entry to 1 of their 5 most-preferred degrees. In this case, they need not list any preferences below the degree for which they have been granted guaranteed entry.

15 American school applicants often have a local ‘district school’ to which they have priority for entry. ‘District School Bias (DSB)’ occurs where an applicant lists her district school above other schools that are in fact more preferred (1). The Boston Mechanism encourages DSB. There is also evidence of higher rates of DSB in other commonly used mechanisms, where, as in the UAC mechanism, the length of ordered lists is constrained (13).

16 There are other ways to overcome this challenge (15).

17 See Tables S1–S5 (Supplementary Material) in the Online Appendix for raw data on observed manipulation classified according to demographic information, namely gender, high school type, sibling order, and prior advice. This information is formally captured by the OLS regression analysis later in this section.
