# Supplementary material for: A field evaluation of a matching mechanism: University applicant behavior in Australia
Source: PNAS Nexus. 2022 Mar 9;1(1):pgac010. doi: 10.1093/pnasnexus/pgac010 (PMC9802117; doi:10.1093/pnasnexus/pgac010)
Supplement: pgac010_Supplemental_File [file pgac010_supplemental_file.pdf]

Supplementary Information for

A Field Evaluation of a Matching Mechanism:University Applicant Behaviour in Australia

Pablo Guillen[a], Onur Kesten[a, *], Alexander Kiefer[a], Mark Melatos[a]

[a] School of Economics, The University of Sydney, Australia.
* To whom correspondence may be addressed. Email: onur.kesten@sydney.edu.au.

This PDF file includes:

Summary Statistics Tables S1 to S5
Experimental Materials

Summary statistics

Table S1: Gender Summary Statistics

	Observations	*GEB* (%)	Include Degree 6 (%)	Any Sub-Optimal Ordering (%)
Male	310	51.9	40.3	70.6
Female	512	57.8	42.9	78.1

The proportions reported in the table relate to the proportion of each gender submitting preferred degree lists that exhibit *GEB*, Include Degree 6 or Any Sub-Optimal Ordering. Note that an applicant's preferred degree list may exhibit more than one of these types of sub-optimal ordering.
The summary statistics for the gender responses 'Other' and 'Prefer Not to Say' are excluded from this table since there were only 2 observations for each response.

Table S2: School Type Summary Statistics

**School Type**	**Observations**	***GEB* (%)**	**Include Degree 6 (%)**	**Any Sub-Optimal Ordering (%)**
**Government Non-Selective**	285	61.8	48.4	82.1
**Government Selective**	186	51.6	45.7	63.4
**Private**	355	53.2	44.5	76.3

The proportions reported in the table relate to the proportion of participants attending each school type submitting preferred degree lists that exhibit *GEB*, Include Degree 6 or Any Sub-Optimal Ordering. Note that an applicant's preferred degree list may exhibit more than one of these types of sub-optimal ordering.

Table S3: Sibling Effects Summary Statistics

**Birth order of participants**	**Observations**	***GEB* (%)**	**Include Degree 6 (%)**	**Any Sub-Optimal Ordering (%)**
First	445	56.0	44.5	74.4
Second	252	52.3	48.4	76.6
Third	87	56.3	40.2	71.2
Fourth	30	76.7	63.3	90.0
Fifth	12	66.7	58.3	83.3

The proportions reported in the table relate to the proportion of participants of each birth order submitting preferred degree lists that exhibit *GEB*, Include Degree 6 or Any Sub-Optimal Ordering. Note that an applicant's preferred degree list may exhibit more than one of these types of sub-optimal ordering.

Table S4: Prior Advice Summary Statistics

	**Observations**	**GEB (%)**	**Include Degree 6 (%)**	**Sub-optimal (%)**
UAC Website	622	55.0	45.5	74.0
UAC Publications	232	53.0	50.9	72.0
University Websites	319	55.2	50.2	74.3
University Open Days	341	55.7	48.7	77.1
Careers Advisors	593	55.5	47.9	74.9
Teachers	547	55.8	45.7	75.3
Parents	187	52.9	43.3	73.3
Siblings	142	54.9	45.8	73.9
Extended Family	44	45.5	36.4	75.0
Friends	270	52.6	47.0	72.6
Other	16	56.3	31.3	75.0

The proportions reported in the table relate to the proportion of participants accessing each source of advice submitting preferred degree lists that exhibit *GEB*, Include Degree 6 or Any Sub-Optimal Ordering. Note that an applicant's preferred degree list may exhibit more than one of these types of sub-optimal ordering.

Table S5: Demographic Variable Regression Coefficients

**Demographic Variables**	**Any Sub-Optimal Ordering**	***GEB***	**Including Course 6**
School Type (Baseline: Public non-selective)			
Selective School	-0.1730237***	-0.0823239*	-0.0349388
	(0.0436428)	(0.0488622)	(0.0482155)
Private School	-0.0481998	-0.0694261*	-0.0264575
	(0.0328858)	(0.0397025)	(0.0404051)
Sibling Order (Baseline: First Sibling)			
Second Sibling	0.030191	-0.0471175	0.0588088
	(0.0368237)	(0.044335)	(0.0446339)
Third Sibling	-0.0348521	-0.0019748	-0.01626891
	(0.055487)	(0.0626968)	(0.0611067)
Fourth Sibling	0.1428417**	0.2053134**	0.2003278**
	(0.0615024)	(0.0859653)	(0.0943831)
Fifth Sibling	0.0607248	0.0588969	0.1480918
	(0.1138756)	(0.1361704)	(0.1445582)
Gender[4] (Baseline: Male)			
Female	0.0505651	0.0383035	0.0717123 **
	(0.0320839)	(0.0366713)	(0.0363655)
Other	0.2417812***	0.4100679***	0.5117445***
	(0.0530707)	(0.0632907)	(0.0606678)
Prefer Not to Say	0.2130404***	0.4829695***	0.5785651***
	(0.0456316)	(0.0550044)	(0.0563346)

[4] ‘Other’ and ‘Prefer Not to Say’ only had 2 observations each.

Prior Advice			
UAC Website	-0.0499197	-0.0072648	-0.0528443
	(0.0373949)	(0.0451305)	(0.0459851)
UAC Publications	-0.0254402	-0.020941	0.0630596
	(0.0372016)	(0.0421276)	(0.0418656)
University Websites	-0.0035124	0.0046983	0.0620312
	(0.0381329)	(0.0425837)	(0.0426888)
University Open Day	0.0633277*	0.0149999	0.019771
	(.0353479)	(0.0401358)	(0.0404339)
Careers Advisor	-0.0293928	-0.0119612	0.0492471
	(0.0343736)	(0.0401931)	(0.0417442)
Teachers	0.0042787	0.0119029	-0.0177682
	(0.0329857)	(0.0385143)	(0.0392569)
Parents	-0.0073034	-0.0159831	-0.0436285
	(0.0391408)	(0.0464301)	(0.0459264)
Siblings	0.0023231	0.0391805	-0.0229908
	(0.0490336)	(0.0572318)	(0.0571947)
Extended Family	0.0098752	-0.0781001	-0.0966
	(0.0688999)	(0.0778949)	(0.0775775)
Friends	-0.0037184	-0.0258487	0.032548
	(0.0382782)	(0.0432348)	(0.0430943)
Other	0.0036133	0.0097754	-0.1515051
	(0.1042272)	(0.1280365)	(0.1305849)
Location (Baseline: Outside of Sydney)			
Sydney	-0.0453206	-0.039781	-0.0516568
	(0.0337144)	(0.0411798)	(0.0413119)

Note: Standard errors in parentheses.
Asterisks indicate significance levels: * = p-value<0.10; ** = p-value<0.05; *** = p-value<0.01
The gender responses ‘Other’ and ‘Prefer Not to Say’ only had 2 observations each.

Each cell includes the coefficient on the Treatment Group listed in the row, when the Treatment Group listed in the column is the omitted Treatment Group. a regression is run with the Treatment Group in the column as the baseline group.

Experimental Materials

Item 1: Email sent to prospective participants

Dear UAC applicant,

You are invited to participate in a voluntary, online, anonymous survey run by researchers from the University of Sydney.

The survey questions are about UAC's procedures. You have been invited to participate because you applied to university through UAC.

If you choose to participate, you will receive $5 monetary compensation for completing the survey plus a bonus. The size of the bonus will depend both on your answers and a random factor. However, you will receive no payment if you fail to complete the survey.

Data from the survey will be collected in unidentified form so nobody, including the researchers, will be able to link your answers to your personal identifiers. For that reason, it will not be possible to withdraw from the study once you have completed the survey.

You have until [DATE and TIME] to complete the survey, if you wish to participate.

Note that you are only allowed to complete the survey once.

Please read through the Participant Information Statement for more details about the study.

You are welcome to contact Associate Professor Pablo Guillen, pablo.guillen@sydney.edu.au, if you have any concerns about this study.

If you are willing to participate in the survey, please click on this survey link.

Thank you for your time and consideration.

Best wishes,

Kim Paino

General Manager, Marketing and Engagement

Item 2: Survey Questions

Please read the attached participant information statement before proceeding:

Participant Information Statement

In order to claim your payment for completing the survey, you will be required to provide your mobile phone number upon completion of the survey questions. **You will also need to register for a PayID, linked to a mobile phone number, by 5:00pm on Monday 14 October, if you do not have a PayID before completing the survey. If you do not register for a PayID, we cannot pay you.** For more information on PayIDs and how to register for one, click on the below link that corresponds to your bank. If your bank is not listed, most banks should provide their own information on how to register for a PayID.

https://www.nab.com.au/personal/customer-support/payid

https://www.anz.com.au/ways-to-bank/more/pay-id/

https://www.westpac.com.au/personal-banking/online-banking/making-the-most/new-payments-platform/

https://www.commbank.com.au/digital-banking/pay-id.html

Please read the attached participant information statement before proceeding:

Participant Information Statement

In order to claim your payment for completing the survey, you will be required to provide your mobile phone number upon completion of the survey questions. **You will also need to register for a PayID, linked to a mobile phone number, by 5:00pm on (INSERT DATE), if you do not have a PayID before completing the survey. If you do not register for a PayID, we cannot pay you.** For more information on PayIDs and how to register for one, click on the below link that corresponds to your bank. If your bank is not listed, most banks should provide their own information on how to register for a PayID.

https://www.nab.com.au/personal/customer-support/payid

https://www.anz.com.au/ways-to-bank/more/pay-id/

https://www.westpac.com.au/personal-banking/online-banking/making-the-most/new-payments-platform/

https://www.commbank.com.au/digital-banking/pay-id.html

You have until 5:00pm on (INSERT DATE), to complete the survey. You cannot complete the survey more than once. If you start, but then close the survey without finishing it, you will need to start again in order to complete the survey.

By answering 'Yes' to this question, you are consenting to taking part in this study. Specifically, you are telling us that you have understood the contents of the attached Participant Information Statement, you agree to take part in the research study outlined in the Participant Information Statement, and you agree to the use of your personal information as described in the Participant Information Statement. If you answer 'No' to this question, you will not be able to proceed to the survey.

Do you consent to taking part in the study?

Yes

No

In this task, you will go through a process very similar to applying for a course through the Universities Admissions Centre (UAC).

For the purpose of this task, you have been awarded an ATAR of 80.00. In the table below, six courses are listed that you can apply for. You are able to apply for a maximum of 5 courses, by submitting a preference list, starting with the course you wish to be considered for first, just as you would have done when applying through UAC.

Based on your preference list, a procedure simulating that used by UAC will be used to determine whether you will be made an offer to a course, and if so, which one. The greater the distance above your ATAR that a Lowest Selection Rank is, the lesser your chance of receiving an offer.

If you are made an offer to a course, you will be eligible to be paid the amount of money listed in the corresponding row of the 'Amount Paid if Made an Offer' column, in addition to the $5 participation fee.

Two pieces of information are included in the table:

Column 1 includes the 'Lowest Selection Rank' for each course. This is the lowest selection rank required to receive an offer to each course in the main Year 12 offer rounds, last year. They can be used as a guide for this year. The lowest selection ranks for courses in a particular year are only known after all offers for that year are made.

Column 2 includes the 'Amount paid if made an offer' for each course. This is the amount you will receive, in addition to the $5.00 participation fee, if you are made an offer to a particular course.

A reminder, that your allocated ATAR for the purpose of this task is 80.00. Also note that you have Guaranteed Entry to course 73207. This means that the university which offers Course 73207 has informed you that your ATAR is above the selection rank cutoff that they will apply to Course 73207.

Based on the information given, submit preferences as if you were applying for courses through UAC.

UAC gives the following advice: "List your 'dream preference' at number one but follow that with realistic preferences. At the bottom of the preference list you should include one or two 'safe' options to ensure that you get an offer."

The university which runs course 73207 gives the following advice: "To be offered a place in a guaranteed entry course, list the course as your first preference when you apply."

Course	Lowest Selection Rank	Amount Paid if Made an Offer
42055	85.00	$10.00
19959	82.00	$9.00
56769	81.00	$8.00
65028	80.50	$7.00
73207 (Guaranteed Entry)	79.00	$6.00
82747	78.00	$5.00

Number a maximum of 5 boxes below, in the order that you want to be considered for them. 1, for the course you want to be considered for first. 2, for the course you want to be considered for next, and so forth.

- [ ] 73207
- [ ] 65028
- [ ] 19959
- [ ] 56769
- [ ] 42055
- [ ] 82747

What is your gender?

- Male
- Female
- Other
- Prefer not to say

What type of school did you attend?

- Public non-selective
- Public selective
- Private

What is your sibling order in your family?

- First
- Second
- Third
- Fourth
- Fifth or higher

What was your home postcode when you applied to UAC?

From which of the following sources did you receive advice about applying to UAC? (Choose all that apply)

- UAC's Website
- UAC Publications
- University Websites
- University Open Days
- Careers Advisor
- Teachers
- Parents
- Siblings
- Extended Family
- Friends
- Other

Are you currently studying at university?

- Yes
- No

Based on the matching task, you are eligible to receive $14.00 for completing the survey.
To claim your payment, please enter your mobile phone number below.

Item 3: Participant Information Statement

THE UNIVERSITY OF SYDNEY

School of Economics
Faculty of Arts and Social Sciences

ABN 15 211 513 464

Pablo Guillen Alvarez
Associate Professor

Room 536
Social Sciences A02
The University of Sydney
NSW 2006 AUSTRALIA
Telephone: +61 2 9036 9188
Facsimile: +61 2 9351 4341
Email: pablo.guillen@sydney.edu.au
Web: http://www.sydney.edu.au/

A survey on UAC procedures

PARTICIPANT INFORMATION STATEMENT

(1) What is this study about?

You are invited to take part in a voluntary, online, anonymous survey about the procedures used by the Universities Admissions Centre (UAC).

You have been invited to participate because you recently applied to university through UAC. This Participant Information Statement tells you about the research study. Knowing what is involved will help you decide if you want to take part in the research. Please read this statement carefully and ask questions about anything that you don't understand or want to know more about.

Participation in this research study is voluntary.

By giving your consent to take part in this study you are telling us that you:

- ✓ Understand what you have read.
- ✓ Agree to take part in the research study as outlined below.
- ✓ Agree to the use of your personal information as described.

(2) Who is running the study?

The study is being carried out by the following researchers:

- Mr Alexander Kiefer, Honours student in the University of Sydney School of Economics;
- Associate Professor Pablo Guillen Alvarez, University of Sydney School of Economics;
- Associate Professor Mark Melatos, University of Sydney School of Economics.

Alexander Kiefer is conducting this study as the basis for the Bachelor of Economics (Honours) at The University of Sydney. This will take place under the supervision of Associate Professor Pablo Guillen Alvarez.

(3) What will the study involve for me?

In the survey you will be asked to provide answers to questions related to the UAC procedures.

(4) How much of my time will the study take?

We don’t expect the survey to take more than 15minutes of your time.

(5) Who can take part in the study?

This study is limited to people who applied to universities through UAC for entry in 2019

(6) Do I have to be in the study? Can I withdraw from the study once I've started?

Being in this survey is completely voluntary and you do not have to take part. Your decision whether to participate will not affect your current or future relationship with the researchers or anyone else at The University of Sydney or UAC.

If you decide to take part in the survey and then change your mind before finishing you don’t need to submit your answers. Incomplete survey data will not be analysed. You will not receive any payment if you fail to submit a complete survey.

The survey software generates unidentifiable data so it will not be possible to withdraw once you have submitted your answers.

(7) Are there any risks or costs associated with being in the study?

Aside from giving up your time, we do not expect that there will be any risks or costs associated with taking part in this study.

(8) Are there any benefits associated with being in the study?

You will receive a $5 monetary compensation for completing the survey plus a bonus. The exact size of the bonus depends on both your answer and a random draw. Payment will require you to provide your mobile telephone number which will be deleted after payment is made.

(9) What will happen to information about me that is collected during the study?

By providing your consent, you are agreeing to us collecting unidentifiable personal information about you for the purposes of this research study. Your information will only be used for the purposes outlined in this Participant Information Statement, unless you consent otherwise.

Note that you will need to provide your name and a mobile telephone number to generate a receipt for the purpose of payment. This information is not part of the survey and will not be matched to your survey answers.

(10) Can I tell other people about the study?

Yes, you are welcome to tell other people about the study.

(11) What if I would like further information about the study?

When you have read this information, Associate Professor Pablo Guillen will be available to discuss it with you further and answer any questions you may have. If you would like to

know more at any stage during the study, please feel free to contact Pablo by email at pablo.guillen@sydney.edu.au.

(12) Will I be told the results of the study?

You have a right to receive feedback about the overall results of this study. Once the study is complete UAC will send an email to all participants including a lay summary of the results.

(13) What if I have a complaint or any concerns about the study?

Research involving humans in Australia is reviewed by an independent group of people called a Human Research Ethics Committee (HREC). The ethical aspects of this study have been approved by the HREC of The University of Sydney (Protocol Number 2019/xxx). As part of this process, we have agreed to carry out the study according to the National Statement on Ethical Conduct in Human Research (2007). This statement has been developed to protect people who agree to take part in research studies.

If you are concerned about the way this study is being conducted or you wish to make a complaint to someone independent from the study, please contact the University using the details outlined below. Please quote the study title and protocol number.

The Manager, Ethics Administration, The University of Sydney:

- **Telephone:** +61 2 8627 8176
- **Email:** ro.humanethics@sydney.edu.au
- **Facsimile:** +61 2 8627 8177

This information sheet is for you to keep
